# Evaluation of MRI/Ultrasound Fusion-Guided Prostate Biopsy Using Transrectal and Transperineal Approaches

**DOI:** 10.1155/2017/2176471

**Published:** 2017-09-28

**Authors:** Susanne Tewes, Inga Peters, Ansgar Tiemeyer, Matti Peperhove, Dagmar Hartung, Stefanie Pertschy, Markus A. Kuczyk, Frank Wacker, Katja Hueper

**Affiliations:** ^1^Department of Diagnostic and Interventional Radiology, Hannover Medical School, Carl-Neuberg Str. 1, 30625 Hannover, Germany; ^2^Department of Urology and Urologic Oncology, Hannover Medical School, Carl-Neuberg Str. 1, 30625 Hannover, Germany

## Abstract

**Purpose:**

To evaluate transrectal (TR) and transperineal (TP) approaches for MRI/ultrasound (MRI/US) fusion-guided biopsy to detect prostate cancer (PCa).

**Materials and Methods:**

154 men underwent multiparametric MRI and MRI/US fusion-guided biopsy between July 2012 and October 2016. 79/154 patients were biopsied with a TR approach and 75/154 with a TP approach. MRI was retrospectively analyzed according to PI-RADS version 2. PI-RADS scores were compared with histopathological results. Descriptive statistics, accuracy, and negative and positive predictive values were calculated. Histopathological results of first, second, and third MRI targeted biopsy cores were compared to evaluate the impact of one verus multiple targeted cores.

**Results:**

Detection rates of PCa were 39% for TR biopsy and 75% for TP biopsy. Sensitivity/specificity for tumor detection with PI-RADS ≥ 4 were 81/69% for TR biopsy and 86/84% for TP biopsy. In 31% for TR biopsy and 19% for TP biopsy, PCa was found in the second or third MRI targeted biopsy core only.

**Conclusion:**

MRI/US fusion-guided biopsy may be conducted with the TR as well as the TP approach with high accuracy, giving more flexibility for diagnosis and the option for focal treatment of PCa.

## 1. Introduction

Over the last ten years, multiparametric MRI (mpMRI) of the prostate has gained rising importance in the diagnosis of prostate cancer (PCa). With mpMRI, high accuracy for the detection of PCa has been reported [1, 2]. PCa detection rates by mpMRI range from 80 to 100% for Gleason score (GS) > 7, from 63 to 97% for GS 7, and from 21 to 75% for GS 6 tumors [3]. MRI/ultrasound (MRI/US) fusion software systems have been evolved and tested over the last few years [4]. Increased detection rates for high-risk PCa and decreased detection rates of low-risk PCa compared to the standard transrectal ultrasound- (TRUS-) guided biopsy method have been reported [5–9]. However, there are also studies showing that MRI-guided biopsy particularly improved detection of significant cancer after previous negative biopsy [8, 10]. International guidelines recommend TRUS-guided biopsy as standard of care for first-round biopsy, while MRI-guided biopsy could be useful in the repeat biopsy setting [11, 12].

Different systems for MRI/US image fusion are available, which allow for transrectal (TR) or transperineal (TP) biopsy or for both [4, 13]. Still, there are no clear recommendations with regard to the TR or TP approach. For systematic TRUS-guided biopsy, cancer detection rates are comparable for both approaches [14, 15].

With regard to complications, with TR prostate biopsy, the incidence rate of infection and rectal bleeding is higher [16] and lesions located in the anterior part of the prostate, particularly in high volume prostates, might be missed. With TP prostate biopsy, on the other hand, the incidence of perineal swelling is higher [16], and, usually for ensuring more patient comfort, it needs to be done in general anesthesia, holding its own risks.

Furthermore, there are still no clear recommendations if more than one targeted biopsy core per PCa suspect MRI lesion significantly increases the cancer detection rate. A recent study by Schimmoller et al. found only minor benefit when taking a second targeted biopsy core in a cohort of 290 patients who underwent in-bore MRI-guided prostate biopsy [17].

The purpose of our study was to evaluate MRI/US fusion-guided biopsy using the TP or the TR approach with the same fusion system in terms of diagnostic accuracy, PCa detection rates, and feasibility in a clinical setting.

## 2. Materials and Methods

### 2.1. Patients

Between July 2012 and October 2016, 154 patients with clinical suspicion of PCa underwent MRI/US fusion-guided biopsy of the prostate. Between July 2012 and January 2015, only the TR approach was performed and, until then, 62 patients underwent fusion-guided biopsy. Therefore, 40% of patients from our cohort underwent TR biopsy, irrespective of prostate size, tumor localization according to MRI, negative initial biopsies, or patients' preferences due to unavailability of the TP approach. Since February 2015, both biopsy approaches were performed at our institution. Since then, detailed information and counseling on risks and advantages of the TR and TP approaches were given to the patients under consideration of prostate size, tumor localization according to MRI, negative initial biopsies, and patients' general condition. Under consideration of this information and patients' preferences, 17 patients underwent TR biopsy and 75 patients underwent TP biopsy after February 2015. This retrospective study focuses particularly on a descriptive analysis of clinical parameters in association with the different biopsy settings. In total, 79/154 patients underwent TR biopsy and 75/154 patients underwent TP biopsy. Written informed consent was obtained from each patient for clinically indicated mpMRI and MRI/US fusion-guided biopsy. The local ethical board waived the requirement for obtaining informed consent for this retrospective analysis. 125 patients had at least one negative previous biopsy (1–7 prebiopsies), 16 patients had no previous biopsy, 11 patients were on active surveillance, and in two patients it was unknown whether a previous biopsy had been performed. Patients' characteristics are summarized in Table 1.

### 2.2. Multiparametric MRI

Multiparametric MRI was acquired according to European Society of Urogenital Radiology (ESUR) guidelines [2, 18] on a 3 Tesla system (MAGNETOM Skyra or MAGNETOM Verio, Siemens Healthcare, Erlangen, Germany) using a 6-channel or 18-channel body coil and a spine coil. In order to reduce bowel movement, all patients without contraindications received an intravenous injection of 20 mg butylscopolamine (Buscopan 20 mg, Boehringer Ingelheim, Germany) prior to the examination. T2 turbo spin echo (TSE) sequences were acquired in transverse, sagittal, and coronal orientation. For diffusion-weighted imaging (DWI), three *b*-values = 0–50, 400–600, and ≥800 s/mm^2^ were used. Dynamic contrast-enhanced (DCE) images were acquired in transverse plane after injection of 0.1 mmol/kg body weight gadoterate meglumine (Dotarem, Guerbet, Aulnay-sous-Bois, France) at a rate of 3 ml/s using a FLASH 3D sequence with an image update rate of 9 s. For details of MRI protocol, please see supplementary Table 1 in Supplementary Material available online at https://doi.org/10.1155/2017/2176471.

Target lesions for MRI/US fusion-guided biopsy were chosen in a clinical setting by experienced uroradiologists. Before the publication of PI-RADS version 2 by the American College of Radiology (ACR), ESUR, and AdMeTech Foundation in December 2014, lesions were scored according to PI-RADS version 1 [18]; since January 2015, lesions were scored according to PI-RADS version 2 [19]. In order to homogenize data for this study, target lesions were retrospectively analyzed according to PI-RADS version 2 [19] and ADC values, prostate volume, and lesion size were documented. Lesion location was reported according to the sector map as suggested in the PI-RADS version 2 document [19].

### 2.3. MRI/US Fusion-Guided and Targeted Biopsy

MRI/US fusion-guided biopsy was performed with the BioJet™ fusion system and software (D&K Technologies, Barum, Germany). The technical data and usage of this system have been described previously [20, 21]. In brief, contouring of the prostate margins and the target lesions was done by a radiologist using the transverse T2 TSE-images. Organ contours were fused with real-time TRUS during the biopsy session.

Patients were informed about advantages and risks of TR and TP prostate biopsy. Indication for TR or TP biopsy was adjusted to the clinical setting under consideration of technical availability, localization of lesion, patients' profile, and patients' preferences.

TR and TP prostate biopsies were performed by two experienced urologists in the dorsal lithotomy position under antibiotic prophylaxis. Local anesthesia (periprostatic block) was performed in patients with a TR biopsy course and general anesthesia was performed for the TP approach. TR MRI/US fusion was performed using a 3D triplane TR ultrasound system (BK Medical, Analogic Ultrasound Group, Pro Focus, Transducer 8818, 9 MHz) solely operating with the side-fire function. An endocavity biplane transducer (BK 8848, 9 MHz, BK Medical, Analogic Ultrasound Group) was used for the TP approach. Biopsy cores with a core length of 22 mm were numbered according to the radiological anatomic sector map as described in the PI-RADS version 1 [18] or version 2 [19] document. The mean number of biopsy cores with the TR approach was 8 ± 3 (range: 4–12) cores per patient with 3 ± 1 (range: 1–7) cores from targeted biopsy and 5 ± 4 (range: 0–11) cores from additional random biopsy.

Using the TP setting, the mean number of biopsy cores per patient was 12 ± 1 (range: 5–12) cores with 3 ± 1 (range: 1–6) cores from targeted biopsy and 8 ± 2 (range: 2–11) cores from additional random biopsy.

**Figure 1:**
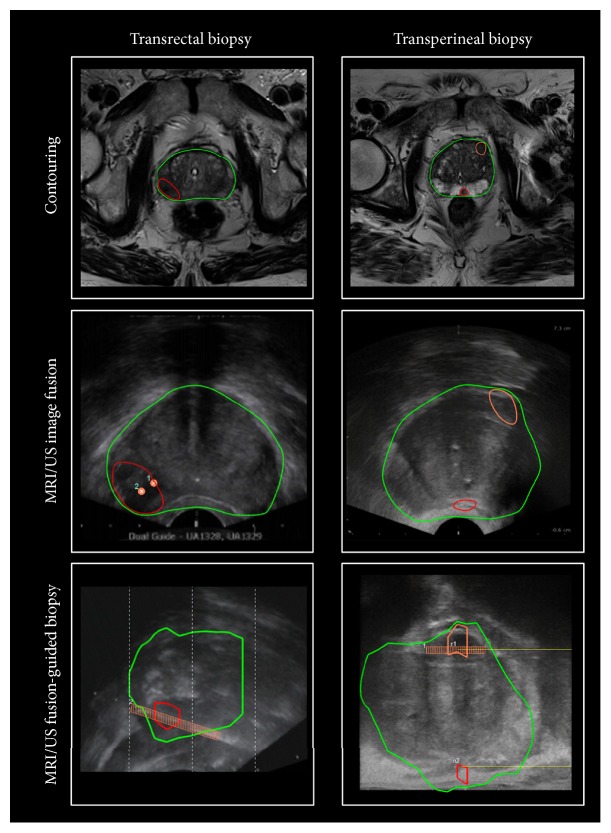
*Workflow of MRI/TRUS fusion-guided biopsy with the BioJet software*. Depicted are two examples of MRI/TRUS fusion-guided biopsy, one with the transrectal (left column) approach and one with the transperineal (right column) approach. First row: contouring of the prostate margins (green) and target lesions (red and orange). Second row: fusion of MRI contours and ultrasound images in the transverse orientation. Third row: obtaining samples from the target lesion. Fusion of MRI contours and ultrasound images are shown in the sagittal orientation. Biopsy cores are marked in orange.

For each cancer-positive biopsy core, a pathologist determined the Gleason grade and Gleason score (GS). Clinically significant cancer was defined as GS ≥ 7 and/or PSA ≥ 10 *μ*g/L, designated as intermediate and/or high-risk tumor groups according to the D'Amico criteria [22]. Examples of MRI/TRUS fusion-guided biopsy with the TR and TP approaches are shown in Figure 1.

### 2.4. Statistical Analysis

For statistical analysis, GraphPad Prism software version 6 (GraphPad Software, Inc., USA) and SPSS software version 24 (IBM Corporation, USA) were used. Clinical data of patients with and without biopsy-proven PCa as well as patients with TR and TP biopsy approaches were compared using unpaired *t*-tests and values are given as mean ± standard deviation (SD). Descriptive analysis, sensitivity, specificity, and negative predictive value (NPV) and positive predictive value (PPV) of mpMRI in TR and TP cohorts for a PI-RADS score ≥ 4 were evaluated with respect to the dominant lesion in each patient and analysis was performed on a per patient basis. The combined histological result of MRI/US fusion-guided and random biopsy cores served as reference.

## 3. Results

### 3.1. Transrectal MRI/US Fusion-Guided Prostate Biopsy (TR Cohort)

In our TR cohort, 31/79 patients were diagnosed with PCa (39%) between July 2012 and September 2016. 60/79 patients had untargeted negative prebiopsies, 6 patients were under active surveillance, 12 patients were biopsy-naïve, and in one patient the prebiopsy status was unknown. One patient was excluded from the analysis because the target lesion located in the anterior part of the prostate could not be reached by TR biopsy due to restriction of the needle guide (at that time point, a TP biopsy approach was technically not available at our institution yet). 664 biopsy cores were taken in total: 271 cores were from targeted biopsy and 393 cores were from additional systematic random biopsies.

PI-RADS scores were significantly higher in patients with PCa compared to patients without PCa (4.1 ± 0.8 versus 2.9 ± 1.0, *p* < 0.001) and ADC values were significantly lower in tumor-positive lesions (0.8 ± 0.2 versus 0.9 ± 0.3 10^−3^ mm^2^/s, *p* < 0.01). Patients with PCa were significantly older (67 ± 8 versus 63 ± 7 years, *p* < 0.05) and prostate volume was significantly lower in the group with PCa (48 ± 26 versus 67 ± 32 ml, *p* < 0.01). No significant difference was found in PSA levels (12 ± 16 versus 8 ± 4 *μ*g/l), lesion size (14 ± 6 versus 12 ± 5 mm), or body mass index (BMI 28 ± 5 versus 27 ± 4 kg/m^2^).

According to the sector map, 27/79 (33%) target lesions from TR biopsy were located in the anterior half of the prostate; 52/79 (66%) target lesions were in the posterior half. Concordantly, the tumor detection rate was slightly but not significantly lower in the anterior (12/31, 39%) than in the posterior prostate (19/31, 61%; Table 2), which is presumably solely the result of lower sampling rates in the anterior gland in this cohort. The detection rate, when only considering the samples in the respective region, was 12/27 (44%) for the anterior gland, while it was 19/52 (37%) for the posterior gland.

In our TR cohort, sensitivity and specificity of 81% and 69% were achieved for mpMRI with a Youden-selected cut-off value of PI-RADS ≥ 4 (area under the curve (AUC) = 0.81 (95% confidence interval (CI): 0.7–0.9)). NPV was 85% and PPV was 63%.

26 out of 31 PCa positive biopsies were obtained by targeted biopsy (Table 3). The cancer detection rate was improved from 33% to 39% when additional random biopsy cores were considered: in five patients (6%), PCa was only found in systematic biopsy (one patient with GS 3 + 3 = 6, one patient with GS 3 + 4 = 7a, one patient with GS 4 + 3 = 7b, one patient with GS 4 + 4 = 8, and one patient with 4 + 5 = 9). A retrospective reanalysis revealed that in two of these five patients no suspicious MRI lesion could be identified in the documented region of the prostate (GS: 3 + 3 = 6 and 4 + 3 = 7b). In the other three cases, the suspicious lesion had been identified at MRI (PI-RADS score 4 in all of the three cases), indicating that MRI/US fusion or the fusion-guided biopsy might have failed (GS: 3 + 4 = 7a, 4 + 4 = 8, and 4 + 5 = 9). Furthermore, in 5/26 patients, additional systematic biopsy cores resulted in a GS upgrade (three patients from GS 3 + 4 = 7a to 4 + 3 = 7b, one patient from GS 4 + 3 = 7b to 4 + 4 = 8, and one patient from GS 4 + 3 = 7b to 4 + 5 = 9; Table 3). A retrospective reanalysis showed that in one of these patients (GS upgrade from 4 + 3 = 7b to 4 + 5 = 9) initial clinical MRI report and choice of index lesion failed and the region of the prostate that revealed highest Gleason score was most suspect on MRI. In the other four cases, systematic biopsy cores that revealed Gleason score upgrade were taken from a region in the prostate that was close to the suspect lesion on MRI and might have contained prostate tissue that appeared suspect on MRI and might have been part of the index lesion.

A subgroup analysis of patients with PCa positive targeted biopsy (*n* = 26) revealed that in 18/26 patients PCa was detected with the first biopsy core, in 7/26 with the second biopsy core, and in 1/26 with the third biopsy core. Thus, taking multiple biopsy cores from the target lesion improved cancer detection from 23% with one biopsy core to 32% with two and 33% with three biopsy cores using the TR approach.

### 3.2. Transperineal MRI/US Fusion-Guided Prostate Biopsy (TP Cohort)

In the TP cohort, 56/75 patients were diagnosed with PCa (75%) between February 2015 and September 2016. 65/75 patients had untargeted negative prebiopsies, five patients were under active surveillance, four patients were biopsy-naïve, and in one patient the prebiopsy status was unknown. 865 biopsy cores were taken: 242 cores were from targeted biopsy and 623 cores from additional systematic biopsies.

PI-RADS scores were significantly higher in patients with PCa compared to patients without PCa (4.4 ± 0.7 versus 3.2 ± 0.4, *p* < 0.001) and ADC values were significantly lower in tumor-positive lesions (0.7 ± 0.2 versus 0.8 ± 0.2 10^−3^ mm^2^/s, *p* < 0.05). Patients with PCa were significantly older (69 ± 8 versus 62 ± 7 years, *p* < 0.001) and prostate volume was significantly lower in the group with PCa (60 ± 40 versus 84 ± 52 ml, *p* < 0.05). No significant difference was found in PSA levels (17 ± 19 versus 13 ± 13 *μ*g/l), in lesion size (18 ± 7 versus 14 ± 9 mm), or in BMI (26 ± 3 versus 28 ± 5 kg/m^2^).

In TP cohort, 59/75 (79%) dominant lesions were located in the anterior prostate and 16/75 (21%) were located in the posterior part of the organ. Concordantly, the PCa detection rate was significantly higher in the anterior gland (44/56, 79%) when compared to the posterior gland (12/56, 21%; *p* < 0.001, Table 2). The cancer detection rate, similar to the TR approach, when only considering the samples in the respective region, was comparable in the anterior gland (44/59, 75%) to the posterior gland (12/16, 75%).

For this cohort, sensitivity and specificity of 86% and 84% were achieved for mpMRI with a Youden-selected cut-off value of PI-RADS 4, with an AUC of 0.89 (95% CI: 0.8–1.0). NPV was 67% and PPV was 94%.

In 54/56 patients with PCa, tumor diagnosis was obtained from targeted biopsy (Table 3). The cancer detection rate was improved from 72% to 75% when additional random biopsy cores were considered: in 2 patients (3%), PCa was only found in the additional systematic biopsy cores (one patient with GS 3 + 3 = 6 and one patient with GS 4 + 3 = 7b). A retrospective reanalysis of these patients revealed that in 1 of these 2 patients no suspicious lesion was detected at mpMRI in the region of PCa (GS 3 + 3 = 6). In the other patient, MRI showed a suspicious lesion (PI-RADS score 5) in the documented region, indicating that MRI/US fusion-guided targeted biopsy failed (GS: 4 + 3 = 7b). Furthermore, in two patients (3%), additional systematic biopsy cores resulted in a GS upgrade (one patient from GS 3 + 3 = 6 to 3 + 4 = 7a and one patient from GS 4 + 4 = 8 to 4 + 5 = 9). A retrospective reanalysis showed that in one patient (GS upgrade from 3 + 3 = 6 to 3 + 4 = 7a) the systematic biopsy core that revealed GS upgrade was taken from a region in the prostate which was close to the suspect lesion in MRI and potentially contained tissue of the index lesion. In the other patient, it is not reproducible from what region in the prostate was the systematic biopsy core with the upgrade retrieved; therefore no reason for upgrading can be identified.

A subgroup analysis of patients with PCa positive targeted biopsy (*n* = 54) revealed that in 44/54 patients PCa was detected with the first biopsy core and in 10/54 patients with the second biopsy core and no additional PCa was found in the third targeted biopsy core. Thus, taking multiple biopsy cores from the target lesion improved cancer detection from 59% with one biopsy core to 72% with two biopsy cores using the TP approach.

### 3.3. Comparison of Clinical Parameters between TR and TP Cohorts

Our TP cohort was significantly older (67 ± 8 versus 65 ± 8 years, *p* < 0.05) and had more previous biopsies (1.6 ± 1.0 versus 1.3 ± 0.9, *p* < 0.05) and higher PSA values (16 ± 19 versus 10 ± 11 *μ*g/l, *p* < 0.05) than patients in the TR cohort. Additionally, PI-RADS scores were higher (4.1 ± 0.7 versus 3.4 ± 1.1, *p* < 0.001) and size of target lesions was larger (17 ± 7 versus 13 ± 5 mm, *p* < 0.001) while ADC values were lower (0.7 ± 0.2 versus 0.9 ± 0.3 10^−3 ^mm^2^/s, *p* < 0.001) than those in our TR cohort. No significant difference in BMI was observed between the two groups (26 ± 4 versus 27 ± 4). Data are summarized in Table 4.

## 4. Discussion

We could demonstrate that mpMRI in combination with MRI/US fusion-guided biopsy yields high detection rates of PCa for both biopsy approaches. The BioJet system provides the opportunity to use both biopsy routes easily and can therefore offer different strategies for the individual patient with respect to the clinical setting. Additional untargeted, random biopsy cores as well as taking a second targeted biopsy core from the target lesion improved tumor detection rates in our study for both biopsy routes.

It is known that, in biopsy-naïve patients, detection rates for PCa with randomized untargeted biopsy are 20–30% [23–25] with decreasing rates of 10–20% for the second biopsy and 5–9% for the third biopsy [25, 26]. However, there are also studies reporting a cancer detection rate of 41% with saturation biopsy (retrieving an average of 24 biopsy cores and more) in patients with previous negative biopsy [27].

In our study, we could achieve cancer detection rates of 39% for TR and 75% for TP combined targeted and systematic biopsies in a patient cohort with primarily prebiopsied patients (90% with at least one previous biopsy), while less biopsy cores were needed than with saturation biopsy: 8 ± 3 cores for TR biopsy (3 ± 1 of which from targeted biopsy) and 12 ± 1 cores for TP biopsy (3 ± 1 from targeted biopsy) were taken.

When using radical prostatectomy tissue as reference, it has been reported that significant PCa might be missed in 8–24% of patients by mpMRI [28–30]. Cancer detection rate in our study was improved from 33% to 39% with TR biopsy and from 72% to 75% with TP biopsy when additional untargeted biopsy cores were considered. It has been stated earlier that the combination of systematic and targeted biopsy schemes provides the highest detection rate [10] and our results go along with that. Nevertheless, in our study, 4 patients (2.6%) showed suspect lesions on MRI but negative results from targeted biopsy, while untargeted biopsy revealed PCa. It can be assumed that either MRI/US fusion or fusion-targeted biopsy has failed in these cases. However, 2.6% in total seems to be a justifiable error with the technique, providing that patients are correctly informed in advance.

Furthermore, evidence suggested that, in MRI/US fusion-guided biopsy, a two-core biopsy should be performed per target lesion [10, 31], but clear recommendations are still missing. However, a recent study found limited benefit when taking a second biopsy core per target lesion with MRI in-bore biopsy [17]. In our TR and TP cohorts, we found PCa diagnosed with an additional targeted biopsy core in about 20–30% of cases, indicating that at least two targeted biopsy cores might be reasonable. A prospective trial would be necessary to elucidate that very important issue.

With the descriptive design of our study (not randomized, different starting points of TR and TP approach, selection bias), comparing the two cohorts is not intended. We could show that, with one software system, both biopsy routes are feasible and equally effective. Our two cohorts differ in some aspects. In 2012, we started using mpMRI and MRI/US fusion-guided biopsy and procedures (MRI protocol, workflow, and reporting) were not standardized due to a lack of recommendations about when to use MRI fusion-guided biopsy in men with clinical suspicion for PCa. Between July 2012 and January 2015, only TR fusion-guided biopsy (*n* = 62) was performed and TP biopsy was introduced in our institution in February 2015. Initial uncertainty about NPV of mpMRI as well as missing recommendation about cut-off values of PI-RADS scores led to higher indication rates of targeted biopsy, even with lower PI-RADS scores in the initial phase of MRI/US fusion-guided biopsy, when we only used the TR biopsy route. This could be an explanation for our finding that, in our patients that underwent TP biopsy (at later time points after February 2015), PI-RADS scores and lesion size were significantly higher and ADC values were significantly lower than those in our patients that underwent TR biopsy predominantly before February 2015. Furthermore, there was an ongoing optimization of MRI protocol and improvement of workflow, personal learning curve in MRI interpretation, and biopsy planning and biopsy performance. An existing learning curve for prostate MRI interpretation has been described [32–34]. Gaziev et al. recently reported that cancer detection rate improved within two years from 42% to 81%, sensitivity/specificity improved from 93/9% to 85/52%, and NPV improved from 67% to 89% [35].

In our study, one anterior lesion could not be reached with TR approach. Therefore, TP biopsy could be helpful to reach the anterior part of the prostate. In our patient cohort, anterior lesions were more often biopsied with the TP approach. Notably, when comparing the yield of PCa detection within the two cohorts, the percentage of detected PCa located in the anterior half of the prostate was related to the percentage of MRI target lesions in the anterior prostate (Table 2). Therefore, the observation of a higher absolute number of anteriorly located PCa in the TP cohort is solely due to the higher absolute number of anteriorly located MRI target lesions and not from a higher detection rate for anteriorly located lesions compared to posteriorly located lesions with the respective biopsy approach.

Previous studies suggested that TR biopsy might hold a higher risk of infection, since faecal bacteria can enter blood circulation after retrieving specimen from the prostate [36]. Particularly in men with higher risks of infection or rectal bleeding (e.g., with hemorrhoids), complications might be avoided with the TP biopsy method. Pain management, on the other hand, is more challenging with TP biopsy, since it has been reported to be more painful [37]. At our institution, TP biopsy is only performed in general anesthesia and therefore might not be suitable for outpatient procedures, although there are few working groups reporting the possibility to obtain it under local anesthesia [38].

Limitations of our study are the retrospective design and solely descriptive analysis of TR or TP biopsy approach in a clinical setting. Patients were not randomized into TR and TP cohort. The decision for one of the two biopsy routes depended on a doctors-patient shared decision, under consideration of patients' preference, prostate size, lesion localization, and technical availability. And, finally, the TP approach has been available since February 2015, which introduced a bias.

## 5. Conclusion

In conclusion, we demonstrate that MRI/US fusion-guided biopsy has high accuracy for the detection of PCa with both TR and TP approaches. Our biopsy system provides the opportunity to offer both biopsy routes to patients at risk of PCa and to adjust diagnostic strategy to the individual clinical setting. In addition, the TP approach gives the options for fusion-guided focal therapy strategies.

## Supplementary Material

Parameters of multiparametric MRI are presented. DCE = dynamic contrast enhancement, DWI = diffusion weighted imaging, FoV = Field of view, TR = repetition time, TE = echo time, TSE = turbo spin echo.

## Figures and Tables

**Table 1:** Patient characteristics.

Number of patients		154
Age (years)	Mean ± SD	66 ± 8
PSA (*μ*g/l)	Mean (range)	13 (0.4–101)
Number of prebiopsies	Mean (range)	1 (0–7)
Prostate volume (ml)	Mean ± SD	63 ± 38
PI-RADS score	Mean ± SD	4 ± 1
Biopsy cores	Total	1529
	Mean ± SD per patient	10 ± 3

PI-RADS: Prostate Imaging Reporting and Data System; PSA: prostate specific antigen; SD: standard deviation.

**Table 2:** Distribution of MRI/TRUS fusion-guided biopsy target lesions and PCa in TR and TP cohorts.

localization	Transrectal		Transperineal	
	All lesions (*n* = 79)	Lesions with PCa^*∗*^ (*n* = 31)	All lesions (*n* = 75)	Lesions with PCa^*∗*^ (*n* = 56)
AFS	9/79 (11.4%)	6/31 (19.4%)	27/75 (36%)	24/56 (42.9%)
Anterior	18/79 (22.8%)	6/31 (19.4%)	32/75 (42.7%)	20/56 (35.7%)
Posterior	52/79 (65.8%)	19/31 (61.2%)	16/75 (21.3%)	12/56 (21.4%)

^*∗*^Lesions with tumor-positive cores after fusion-guided and systematic biopsy. AFS: anterior fibromuscular stroma; PCa: prostate cancer.

**Table 3:** Frequency of detection of low-risk, intermediate, and high-risk PCa with targeted MRI/TRUS fusion-guided and systematic biopsy.

Risk group according to D'Amico criteria	Transrectal		Transperineal	
	Targeted	Systematic	Targeted	Systematic
Low risk	6	1	16	2
Intermediate and high risk	15	9	36	2
All PCa	21	10	52	4

The number of lesions with highest GS detected with targeted and systematic biopsy is given. The overall PCa detection rate is not shown. Highest GS were more frequently detected with targeted than with systematic biopsy with both biopsy routes. PSA: prostate specific antigen.

**Table 4:** Comparison of clinical parameters of transrectal (TR) and transperineal (TP) cohorts.

Clinical and MRI parameters	TR Mean ± SD	TP Mean ± SD	*p* value^*∗*^
Age (years)	65 ± 8	67 ± 8	<0.05
PSA (*μ*g/l)	10 ± 11	16 ± 18	<0.05
Number of prebiopsies	1.3 ± 0.9 (0–4)	1.6 ± 1.0 (0–7)	<0.05
Prostate volume (ml)	58 ± 31	68 ± 45	ns
BMI (kg/m^2^)	27 ± 4	26 ± 4	ns
PI-RADS^*∗∗*^	3 ± 1	4 ± 1	<0.001
ADC (10^−3^ mm^2^/s)	0.9 ± 0.3	0.7 ± 0.2	<0.001
Lesion size (mm)	13 ± 5	17 ± 8	<0.001

^*∗*^Unpaired  *t*-test. PSA: prostate specific antigen; BMI: body mass index; ADC: apparent diffusion coefficient; ns: nonsignificant. ^*∗∗*^PI-RADS (overall score: 1–5): Prostate Imaging Reporting and Data System.

## Abbreviations

ACR: American College of Radiology
ADC: Apparent diffusion coefficient
AFS: Anterior fibromuscular stroma
AUC: Area under the curve
DCE: Dynamic contrast-enhanced imaging
DWI: Diffusion-weighted imaging
ESUR: European Society of Urogenital Radiology
GS: Gleason score
mpMRI: Multiparametric magnetic resonance imaging
NPV: Negative predictive value
PCa: Prostate cancer
PI-RADS: Prostate Imaging Reporting and Data System
PPV: Positive predictive value
PSA: Prostate specific antigen
SD: Standard deviation
TP: Transperineal
TR: Transrectal
TSE: Turbo spin echo
US: Ultrasound.
