# Supplementary material for: Evaluation of MRI/Ultrasound Fusion-Guided Prostate Biopsy Using Transrectal and Transperineal Approaches
Source: Biomed Res Int. 2017 Sep 28;2017:2176471. doi: 10.1155/2017/2176471 (PMC5637860; doi:10.1155/2017/2176471)
Supplement: Supplementary file 1 — Parameters of multiparametric MRI are presented. DCE = dynamic contrast enhancement, DWI = diffusion weighted imaging, FoV = Field of view, TR = repetition time, TE = echo time, TSE = turbo spin echo. [file 2176471.f1.docx]

Supplement Table 1. Parameters of mpMRI.

|  | T2 TSE | DWI | DCE (T1 vibe) |
| --- | --- | --- | --- |
| TR (ms) | >6000 | >4500 | 5 |
| TE (ms) | >60 | >70 | 1.5 |
| FoV (mm²) | 200x200 | 200x200 | 259x259 |
| matrix | 320x320 | 140x140 | 192x192 |
| slice thickness (mm) | 3mm | 3 mm | 3.3 mm |
| b-values (s/mm²) | - | 0-50, 400-600, ­­≥800 | - |

Parameters of multiparametric MRI are presented. DCE=dynamic contrast enhancement, DWI= diffusion weighted imaging, FoV=Field of view, TR=repetition time, TE=echo time, TSE=turbo spin echo.
